# Characterization of small nucleolar RNA retaining transcripts in human normal and cancer cells

**DOI:** 10.1016/j.ncrna.2025.05.004

**Published:** 2025-05-09

**Authors:** Guglielmo Rambaldelli, Sidra Asghar, Giulia Venturi, Federico Zacchini, Margherita Serra, Catia Giovannini, Laura Gramantieri, Marco Bernini, Alberto Inga, Erik Dassi, Lorenzo Montanaro

**Affiliations:** a Department of Medical and Surgical Sciences (DIMEC), Bologna University, Via Massarenti 9, 40138, Bologna, Italy; b Departmental Program in Laboratory Medicine, IRCCS Azienda Ospedaliero-Universitaria di Bologna, Via Albertoni 15, 40138, Bologna, Italy; c Unit of Breast Surgery, IRCCS Azienda Ospedaliero-Universitaria di Bologna, Via Albertoni 15, 40138, Bologna, Italy; d Division of Internal Medicine, Hepatobiliary and Immunoallergic Diseases, IRCCS Azienda Ospedaliero-Universitaria di Bologna, Via Albertoni 15, 40138, Bologna, Italy; e Department of Cellular, Computational and Integrative Biology (CIBIO), Trento University, Via Sommarive 9, Povo, 38123, (TN), Italy

**Keywords:** Intron-retention, Long-read transcriptomic analysis, RNA isoforms, Non-coding RNA

## Abstract

Small nucleolar RNAs are non-coding RNAs typically encoded within the introns of both protein-coding and non-coding genes. Interestingly, a significant fraction of snoRNA sequences is found as retained introns of specific mRNA isoforms expressed from their host gene. In the present study, we aimed to define the representation of small nucleolar RNA retaining transcripts across various human cell types and tissues including cancer. We found that these type of transcripts are widely represented in normal tissues and cancer-derived cell lines, appearing both in their full-length form and, frequently, in a shorter variant. We characterized the shortening position, which occurs at or very close to the retained small nucleolar RNA sequence at the 5′ end. Interestingly, for some transcripts this shorter variant represents the only form detected. In addition, some of the small nucleolar RNA retaining transcripts can be localized into the cellular cytoplasmic fraction. Moreover, our findings point out that a variable but consistent proportion of small nucleolar RNA sequences in cells, tissues, and liquid biopsy samples is, in fact, present as small nucleolar RNA retaining transcripts, indicating that these elements should be carefully considered when snoRNA are evaluated as biomarkers. Considering that short reads and gene-based transcriptomic analysis completely overlooked these transcripts, potentially missing critical insights into their involvement in cancer and other diseases, our results strongly indicate that these type of transcripts should be further investigated in different contexts to better understand their biogenesis, sequence features, presence, and role within cells.

## 1 Introduction

Small nucleolar RNAs (snoRNAs) constitute a class of non-coding RNAs of small-to-middle size, known for their role in guiding site-specific RNA modifications such as 2′-O-methylation and pseudouridylation [1]. SnoRNAs are primarily classified into C/D box and H/ACA box, based on their structure, function and association with two distinct sets of core proteins to form functional small nucleolar ribonucleoproteins (snoRNP) [2]. C/D box snoRNAs (also termed SNORD) associate with SNU13, NOP56, NOP58, and the methyltransferase fibrillarin (FBL) to guide 2′-O-methylation of target ribose residues in RNA. Conversely, H/ACA box snoRNAs (termed as SNORA) associate with NHP2, NOP10, GAR1, and the pseudouridine (Ψ)-synthase dyskerin (DKC1) to guide the isomerization of target uridines to Ψs [3]. SnoRNP-mediated RNA modifications are site-specific, this specificity being dependent on the base pairing between the guide snoRNA and the target sequence. Most residues targeted by snoRNPs are located in ribosomal RNA (rRNA) with a small fraction found in spliceosomal small nuclear RNAs, lncRNAs, miRNAs, tRNAs and mRNAs. These modifications impact post-transcriptional control, influencing gene expression at various levels. Recent findings on alterations in snoRNAs in the context of cancer suggest their significant involvement in the neoplastic process [4]. Numerous studies have demonstrated a correlation between changes in the expression of certain snoRNAs and various cancer characteristics such as proliferation, migration, invasiveness, and their association with patient prognosis [[5], [6], [7], [8]].

In vertebrates, most snoRNA genes are located within intronic sequences of genes involved in the synthesis or function of the translational apparatus. These genes include those coding for ribosomal proteins, translation factors, nucleolar proteins, and proteins involved in mRNA binding, transport, and stability. In certain instances, snoRNAs are embedded within non-coding genes named small nucleolar RNA host genes or SNHG. Regardless of their location, both coding and non-coding host genes are transcribed by RNA Pol II. SnoRNAs are primarily generated through the splicing of nascent pre-mRNA and the exonucleolytic trimming of the spliced intron [[9], [10], [11]]. They are believed to be significantly linked to various diseases, including cancer [12]. Their role in regulating the development and biological behavior of cancer has also been identified with numerous studies highlighting snoRNAs as valuable prognostic and diagnostic markers for cancer [4,13].

**Fig. 1:**
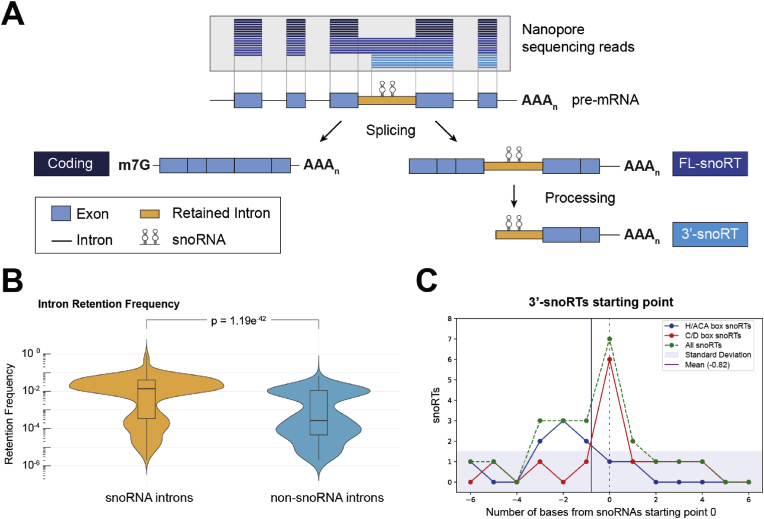
**Detection of snoRTs forms. A**: Schematic representation of pre-mRNA processing and generation of snoRTs, with specific insight on the ONT reads distribution. **B:** Violin plots illustrating the distribution of the retention frequency of snoRNA containing introns (snoRNA introns) compared to all the other introns reported in snoRNA host genes (non-snoRNA introns) in the panel of cell lines considered [14]. Box-plots show median and first and third quartile values. **C**: Distribution of the 3′-snoRTs starting point relative to the snoRNA sequence beginning.

Evidence from our research [14] and other studies [15,16] suggests that during the maturation of snoRNA host-genes, non-canonical processes can lead to the retention of an intron containing the sequence of a snoRNA in a mature host gene transcript. These distinct isoforms are commonly referred to as snoRNA retaining transcripts (snoRTs) [[14], [15], [16]]. We recently determined that some snoRTs could localize in the cytoplasm bound to the core proteins of the modification complex and associate with polysomes and selected mRNAs [14]. Moreover, our observations also indicate that snoRTs can be present in 2 major distinct forms: 1) as full-length mRNA isoforms which retain the snoRNA containing intron (FL-snoRT) and 2) as processed 3′ polyadenylated fragments terminating at their 5′ in proximity to the intronic snoRNA-containing sequence (3′-snoRT) (see Fig. 1A for a graphical representation of snoRTs) [17]. This is consistent with previous findings on SNORD86, a C/D box snoRNA hosted in the gene encoding NOP56, a component of the 2-O′-ribose methylation complex [16]. This study demonstrates that the FL-SNORD86/NOP56 snoRT is initially recruited to polysomes, which then activates an RNA decay mechanism, leading to the formation of the 3′ SNORD86/NOP56 snoRT.

snoRTs have been largely overlooked by gene-based RNA-seq analyses available in public databases and their potential physiological functions have thus been poorly explored. In the present study we aim to define the representation of snoRTs in their different forms across various human cell types and tissues including cancer. This will be achieved by analyzing existing transcriptomic datasets obtained through full-length sequencing approaches, coupled with quantitative determination of selected snoRTs.

## 2 Materials and methods

### 2.1 Analysis of Oxford Nanopore Technology public datasets

Three publicly available RNA-seq datasets [[18], [19], [20]] generated using Oxford Nanopore Technology (ONT) sequencing were reanalyzed for the purpose of this study. A total of 621 snoRNAs were selected from the snoDB [21] database based on the presence of the corresponding annotated transcripts deposited in the Ensembl database v.113 [22]. To identify snoRTs, reads overlapping the snoRNA host gene loci were extracted and subjected to BLAST analysis [23] against a reference RNA sequence of the host snoRNA.

An unpaired T-test has been used to compare the intron retention frequency of the two populations of introns: snoRNA hosting introns against all the other non snoRNA retaining introns of each host genes. Furthermore, the frequency of intron retention has been compared between canonical rRNA targeting snoRNA and non-canonical snoRNA using a chi-square test with Yates’ correction, Canonical snoRNA where defined based on the presence of at least one RNA target on snoDB.

Subsequently, fifty snoRNA host gene transcripts, based on intron retention relative abundance, were manually inspected to identify a conservative threshold for distinguishing between different forms. To systematically identify and quantify snoRT forms, a custom pipeline was developed using the Bash scripting language and several bioinformatics tools: bedtools v2.30.0 [24] for manipulating BED files, samtools v. 1.13 [25] for processing sequencing data in SAM/BAM format, and BLAST v. 2.12.0+ for sequence similarity search. Finally, the data statistical analysis and plotting was performed using R v. 4.4.2.

### 2.2 Cell culture

All the cell lines (MCF7, Snu475, Huh7, MDA-MB-231, HepG2 and ZR-75-1) were obtained from the American Type Culture Collection (ATCC). ZR-75-1, MCF7, Huh7, Snu475 cells were cultured in RPMI 1640 medium supplemented with 10 % FBS, 100 U/mL penicillin, 0.1 mg/mL streptomycin, and 2 mM L-glutamine. MDA-MB-231 and HepG2 cells were grown using DMEM with 10 % FBS, 100 U/mL penicillin, 0.1 mg/mL streptomycin, and 2 mM L-glutamine. Cells were maintained at 37 °C and 5 % CO_2_ and regularly checked for mycoplasma contamination. Once the cells reached 70–80 % confluency they were collected by trypsinization.

### 2.3 RNA extraction and cDNA synthesis

Tumor tissues and plasma samples from patients with breast cancer (BRCA) and hepatocellular carcinoma (HCC) (Supplementary Table 1) were collected at our institution (Approval n. 140/2019/Sper/AOUBo and 528/2021/Sper/AOUBo). Plasma samples from healthy subjects (5 males and 5 females, age range 40–50 years) were obtained from CliniSciences. Human XpressRef Universal Total RNA (Qiagen) containing RNA from normal human tissues from various origin was used to provide a context of general physiological baseline of snoRTs expression in normal human tissue. Total RNA was extracted from cell lines and tissue samples by using the miRNeasy Mini kit (Qiagen) according to manufacturer's guidelines. cDNA was obtained by reverse transcribing 500 ng of RNA using GoScript cDNA synthesis kit (Promega) at 50 °C according to the standard instructions provided by the manufacturer. Blood samples were collected in EDTA vacuum tubes and centrifuged at 2000 RCF for 10 min at 4 °C to separate the plasma. The plasma was then centrifuged at 16000 RCF for 10 min at 4 °C and stored at −80 °C. Total RNA was obtained from 200 μL of plasma using miRNeasy Micro kit (Qiagen) according to the manufacturer's guidelines. Extracted RNA samples were subsequently reverse transcribed into cDNA by using GoScript cDNA synthesis kit (Promega) with random hexamers as previously described.

### 2.4 ddPCR analysis

Digital PCR reactions were performed in QX200TM Droplet DigitalTM PCR System (Bio-Rad). The ddPCR reaction was assembled using ddPCR™ EvaGreen Supermix and specific primers (Supplementary Table 2). cDNA loaded per reaction was different for each sample type (BRCA and HCC tissue 30 ng, cell lines 15 ng, plasma 14 ng). QuantaSoft™ Software was used for absolute quantification of all the targets. For each family of snoRTs, three sets of primers were designed: Full-Length primers specific for FL-snoRT, snoRT primers able to amplify both FL-snoRT and 3′-snoRT, snoRT + snoRNA primers were able to amplify snoRNA, FL-snoRT and 3′-snoRT (see results and discussion section “3.3.1 Quantification of snoRNAs and snoRTs in cell lines” for a graphical example of primers design). Therefore, the following calculations were made to determine the absolute quantities of each isoform: total copies/μL of 3′-snoRT were obtained by subtracting the absolute amount of copies/μl obtained using Full-Length primers to the number of copies obtained using snoRT primers. Total copies/μL of snoRNAs were computed by subtracting the copies/μL obtained with snoRT primers from copies/μL of snoRT + snoRNA primer.

## 3 Results and discussion

### 3.1 Detection of snoRTs forms

snoRTs can either be found in a FL- or in a 3′-shorter form. To obtain a clear picture of their representation, we aimed at quantifying the frequency of the retention of the snoRNA-containing introns and then at developing a method to distinguish these two forms. For this reason, we re-analyzed the RNA sequencing data generated by Ying Chen et al. [18]. These data were selected because they were obtained through ONT technology and characterized by a very high average sequencing depth (34 million long reads per cell line) on five established human cancer cell lines: MCF7 (breast carcinoma), K562 (chronic myelogenous leukemia), HepG2 (hepatocellular carcinoma), HCT116 (colorectal carcinoma), and A549 (lung adenocarcinoma). Notably, snoRNA host genes are known to be highly expressed in cancer cell lines [26].

The primary rationale in choosing ONT sequencing lies in its inherent ability to generate full-length reads. This is particularly advantageous for accurately detecting transcripts of varying lengths, allowing us to explore the different forms of snoRNA retaining transcripts in detail (Fig. 1A). It is important to note that, although ONT allows long-read sequencing, the average read length can vary depending on the dataset, which may introduce limitations in isoform detection. For short 3′-snoRTs, length biases introduced by PCR amplification during ONT library preparation can lead to imprecise quantification of these transcripts [27]. Conversely, some full-length (FL)-snoRT transcripts may exceed the average ONT read length, resulting in inaccurate identification of 3′ and 5′ boundaries [28].

However, since the goal of this analysis is limited to the identification of snoRTs, such quantification inaccuracies do not compromise the main conclusions. Future studies could improve snoRT detection by employing direct RNA or direct cDNA sequencing, or by complementing ONT with sequencing approaches optimized for short transcripts [18]. Additionally, the original library preparation utilized Poly(A) selection, which enabled a high sequencing depth for most snoRTs but limited the detection of canonical snoRNAs and certain known snoRNA 3′-tailed snoRTs [29,30].

To determine whether the retention of snoRNA-containing introns is a specific event rather than a random occurrence of general intron retention (IR) we compared the retention frequency of the snoRNA containing introns (0.006) with that of all the other introns of snoRNA host genes (0.0001), observing a 51.06 fold change difference (p = 1.19e-42). This analysis included all the expressed snoRNA host genes in the dataset and considered each intron retention event individually, regardless of the presence of the snoRNA sequence (Fig. 1B). These findings indicate that the retention of snoRNA-containing introns is a highly frequent event that differs significantly from the general intron retention observed in the studied cellular models. Among the 621 snoRNA genes deposited on ensembl [31], we manually inspected the transcripts from 50 snoRNA host genes selected for the highest percentage of intron retention (Supplementary Table 3). We then aimed at identifying a conservative threshold to distinguish between FL- and 3′-snoRT forms. This revealed three possible scenarios: 1 – the snoRNA-containing intron is retained between two putatively translated exons, 2 – snoRNA retained inside an untranslated region (UTR) of a coding transcript, 3 – snoRNA present inside a non-coding transcript (e.g., SNHG). Among the three categories the majority (25/50) of the analyzed snoRTs belong to the first category. In categories 2 and 3, the presence of highly expressed transcripts overlapping the snoRNAs, combined with the large number of alternative transcripts, made it challenging to reliably determine precise cut points. Focusing on category 1, leveraging ONT long reads that span the complete transcript, a threshold to differentiate between different snoRT forms was identified based on the reads 5′ end. The 5′ end distribution shows that the median cut point aligns precisely with the start of the C/D box snoRNA sequences and occurs two bases upstream of the H/ACA box snoRNA sequences (Fig. 1C). Based on this observation, a conservative threshold was defined as ten nucleotides upstream of the snoRNA gene to differentiate between FL- and 3′-snoRT forms. By applying such threshold snoRTs spanning more than ten nucleotides upstream of the snoRNA start were classified as FL-, while those spanning ten or fewer nucleotides were categorized as 3′-snoRTs. These initial findings indicate that the 3′-form can be detected in several snoRTs retaining both C/D box and H/ACA box snoRNA sequences. This observation is more evident when the snoRNA-containing intron remains within a coding sequence, but it can be generalized considering that the majority of the expressed snoRNA host genes in the considered dataset are actually coding. This aligns with the findings from NOP56–SNORD86 snoRT, indicating that a translation-dependent RNA decay mechanism contributes to the generation of the 3′-form [16].

### 3.2 SnoRTs representation in human cells and tissues

**Fig. 2:**
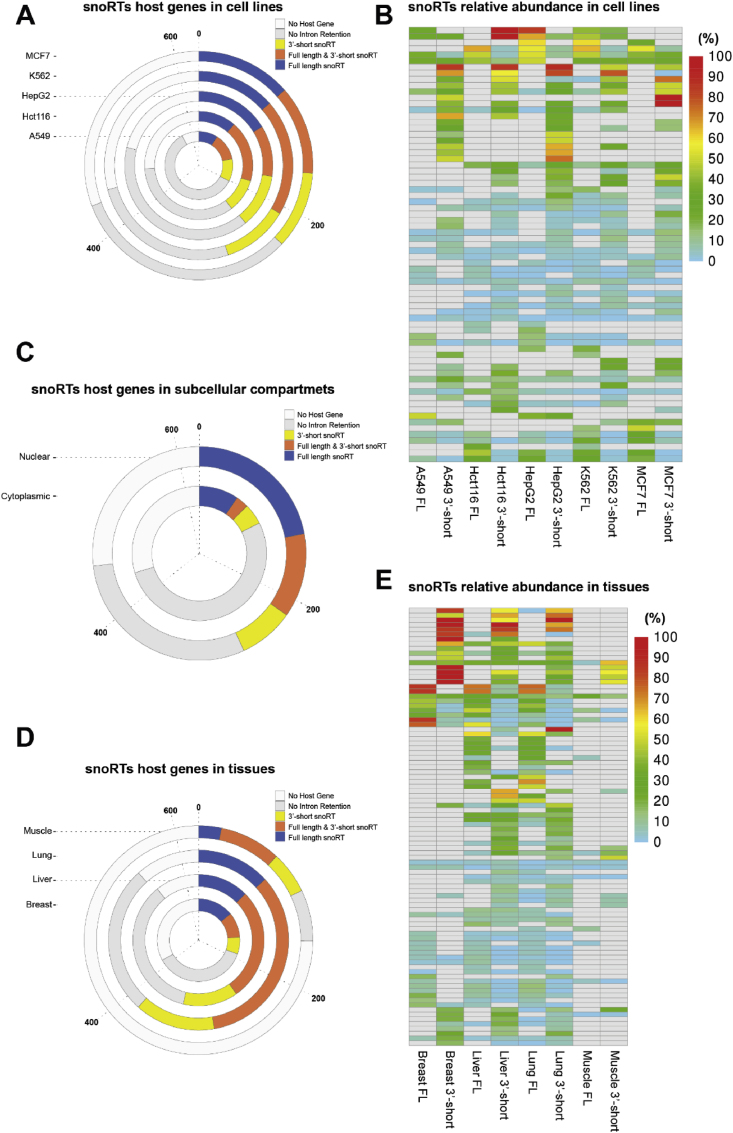
**Global representation of snoRTs in cell lines and tissues. A, C, D:** Distribution of 3′- and FL-snoRTs among the 621 analyzed snoRNA host-genes in the cell lines, nuclear and cytoplasmic compartments and tissues. **B, E:** Heatmaps illustrate the 3′- and FL-snoRTs relative abundance over the total host gene expression in cell lines and tissue, respectively. Only snoRTs for which one of the two forms constitutes at least 1 % or more of the total host gene expression are shown.

We then analyzed the ONT dataset obtained from the cancer-derived cell lines considered above. For each snoRNA host gene, we assessed the presence of reads from transcripts retaining snoRNA sequences. These identified snoRTs were further classified into 3′-snoRTs and FL-snoRTs (Supplementary Table 4) according to the identified threshold, focusing on their abundance (Fig. 2 Panel A). Our findings revealed a widespread presence of both 3′-snoRTs and FL-snoRTs across all the five analyzed cell lines. A complex scenario is suggested by the observation that the presence and the abundance of the different 3′-snoRT and FL-snoRT forms exhibit a degree of dependence on both the specific sequence considered and the cell type (Fig. 2 Panel A–B). Moreover, we expanded our investigation by re-analyzing publicly available sequencing data generated by Oguchi Y. et al. [19] on nuclear and cytoplasmic fractions isolated from the K562 cell line (Fig. 2 Panel C). Data obtained from this study were characterized by a lower sequencing depth (average sequencing depth 1 million long reads per sample) than the ones previously analyzed. Notably, however, the protocol applied for cellular fractioning was carefully validated enabling us to examine the subcellular localization of snoRTs. Interestingly, in line with the observations from us and Lykke-Andersen [14,16] on selected targets, a significant portion of the identified snoRTs was found to be present within the cytoplasmic fraction, in contrast to the expected nuclear localization of canonical snoRNAs. This observation suggests the possibility of a distinct maturation or functional mechanism for these cytoplasmic snoRTs compared to their nuclear counterparts. Coverage and depth limitations are important to consider. Not all the investigated host genes (n = 621) were represented within this last dataset. This likely reflects a combination of factors, including differences in sequencing depth and tissue-specific expression patterns.

To obtain a general picture of the presence and features of snoRTs in a physiological context, a re-analysis of publicly available data generated by Glinos et al. [20] on healthy human tissues was performed on 26 samples from breast, lung, liver, and muscle. This is, to our knowledge, one of the few studies providing full-length transcriptomic data at a considerable depth (average sequencing depth of 6 million long reads per sample) on a substantial number of normal human samples. In this dataset we also observed that the frequency of the snoRNA containing intron retention (0.005) is significantly higher than the retention of all other introns of the expressed snoRNA host genes (0.0002 - FC = 21.87 - p = 2.86e-34) (Supplementary Fig. 1). Moreover, as reported in cell lines, our results revealed a widespread presence of both 3′-snoRTs and FL-snoRTs across all the four analyzed tissues (Fig. 2 Panel D). The sequence and tissue specificity linked to the snoRT representation are even more evident in normal tissues as compared to cell lines (Fig. 2 Panel E). This suggests a potential tissue-specific biological significance for these transcripts, particularly considering the known regulatory roles of snoRNAs in various cellular processes.

Altogether, third-generation transcriptomic analysis on cell lines and tissues derived from healthy donors showed a wide, cell type, and transcript-dependent distribution of snoRTs in both their long and short forms. These findings, however, fail to provide clear functional insights on snoRTs role in the cells. From the functional standpoint, snoRNAs are commonly classified either as canonical, when they are bound to the modification core proteins and guide target RNA modification, or non-canonical, when their target is not identified and/or they play a functional role not involving RNA modification [32]. To obtain preliminary information on the possible role of snoRTs functional relevance we compared the intron retention frequency of the host genes of canonical snoRNAs to that of the non-canonical. This analysis revealed a higher IR frequency in canonical snoRNA than in non-canonical in cell lines (X-squared = 7.528, p = 0.0061) and in normal tissues (X-squared = 9.223, p = 0.0024). Although, the classification of canonical vs non canonical snoRNAs is primarily based on sequence prediction and should therefore be considered with caution, this finding suggests that the functional role of the snoRNAs may contribute in snoRT biogenesis.

### 3.3 snoRNAs and snoRTs presence in human cancer

Considering the well-known dysregulation in snoRNA expression observed in cancer, it appeared interesting to evaluate snoRT expression patterns in samples derived from cancer patients such as cell lines, primary tumor tissues, and plasma. Currently, however, there is a lack of data on full length transcripts from cancer samples at a considerable sequencing depth. In addition, the ONT approach, which typically focuses on polyadenylated transcripts allows to monitor very well the different snoRT forms but fails to assess their quantitative relationship with their correspondent mature snoRNAs. To overcome these limitations in a simple and convenient way we developed a digital droplet PCR assay. It enables the simultaneous quantification of the absolute amount of both the FL- and 3′-forms of selected snoRTs, along with their corresponding mature snoRNA. We therefore selected three snoRTs and investigated their expression in different samples paying particular attention to the relative distribution of the 3′- and FL-forms in comparison to the mature snoRNA. Taking advantage of previously considered ONT data, we selected one H/ACA box snoRT (RPL10-SNORA70) and one C/D box snoRT (RNF149-SNORD89) with a clear and distinguishable separation between the FL- and 3′ snoRTs. Additionally, we evaluated the EIF4A1 snoRTs containing SNORA67 (EIF4A1-SNORA67) that we previously analyzed and for which the nature of both isoforms is known [14].

#### 3.3.1 Quantification of snoRNAs and snoRTs in cell lines

**Fig. 3:**
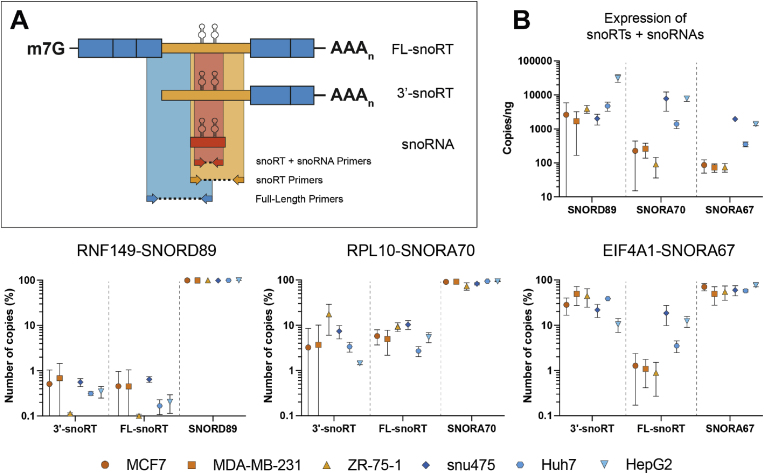
**Quantification of snoRNAs and snoRTs in cancer derived cell lines. A:** Schematic representation of the 3 sets of primers/amplicons designed to identify snoRNA, FL- and 3′-snoRTs. **B:** Violin plots illustrating the absolute abundance of sequences detected using snoRT + snoRNA primers in the 6 considered cell lines (Upper panel). Relative representation of 3′-snoRTs, FL-snoRTs and snoRNAs for each host gene considered. Graphs report median and standard deviation values for each cell line performed on three replicates.

To ensure the distinction between the two isoforms (3′- and FL), we performed a droplet digital RT-PCR (ddPCR) analysis with primers capable of selectively identifying the full-length snoRTs and 3′-snoRTs (Fig. 3 panel A). We applied this approach to a limited series of cancer cell lines derived from two of the tissues considered by the Glinos et al. study [20], i.e. breast and liver. Triplicates from liver cancer cell lines (HepG2, snu475 and Huh7) and breast cancer cell lines (MCF7, ZR-75-1, MDA-MB-231) were used for the purpose of this analysis. The ddPCR results confirmed the presence of all the considered snoRTs and their respective snoRNA in all the tested cell lines. Although the 3′-snoRT and FL-snoRT isoforms of RNF149-SNORD89 were detected at lower levels, those for RPL10-SNORA70 and EIF4A1-SNORA67 were readily detected across cell lines, exhibiting variable levels of abundance (Fig. 3 Panel B).

#### 3.3.2 snoRNAs and snoRTs in human cancer tissues

**Fig. 4:**
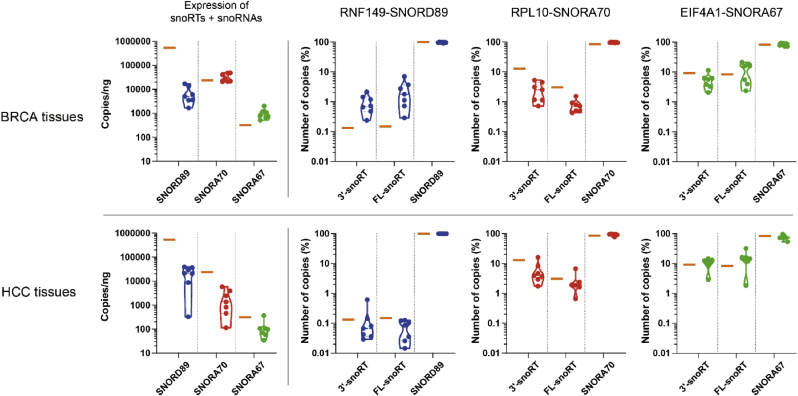
**snoRNAs and snoRTs in human cancer tissues.** snoRTs and snoRNAs evaluation in 7 samples from breast cancer (BRCA – upper panels) and hepatocellular carcinoma (HCC – lower panels). Violin plots illustrate the absolute abundance of sequences detected using snoRT + snoRNA primers (left panels) and the relative distribution of 3′-snoRTs, FL-snoRTs and snoRNAs (right panels) of each analyzed snoRNA sequences. The same analyses are performed on commercial RNA derived from human normal tissues of various origins (orange bars).

After validating the presence of snoRNAs and their respective 3′-snoRT and FL-snoRT in cell lines, we moved towards investigating their abundance in hepatocellular carcinoma (HCC) and breast cancer (BRCA) tissues (Fig. 4). The ddPCR results indicated the presence of significant amounts of SNORD89, SNORA70, and SNORA67 in both BRCA and HCC tissue samples. Additionally, the presence of 3′- and FL-snoRTs were also identified for all three snoRNAs. However, these isoforms were less prevalent for RNF-SNORD89 and RPL10-SNORA70, while both 3′- and FL-snoRT isoforms of EIF4A1-SNORA67 were notably more abundant. The varying abundance pattern of these isoforms among the tissue samples supports the importance of better understanding their presence and biogenesis.

#### 3.3.3 snoRNAs and snoRTs in human plasma

**Fig. 5:**
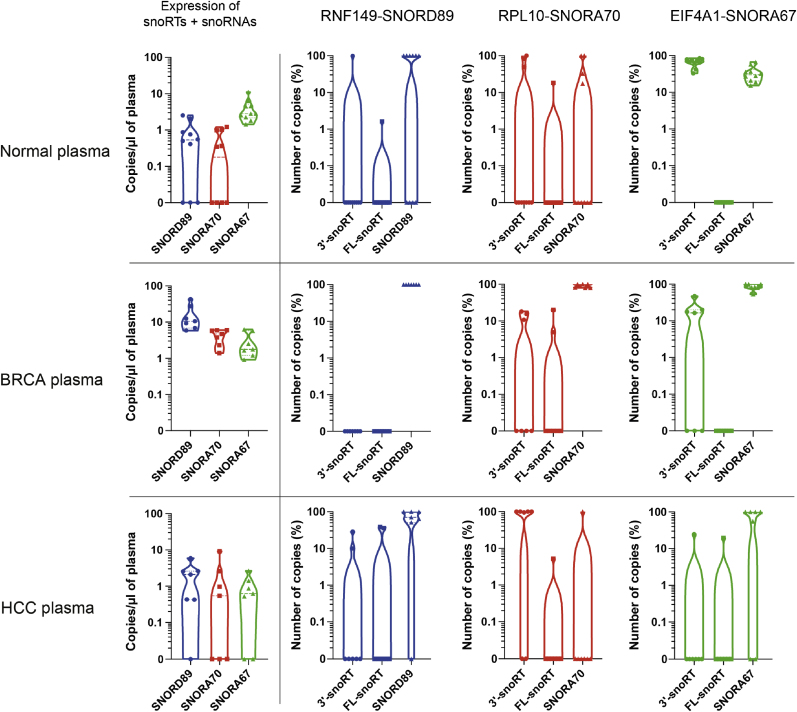
**snoRNAs and snoRTs in human plasma.** snoRTs and snoRNAs evaluation of plasma samples from 10 healthy subjects (Normal plasma – upper panels), 7 breast cancer (BRCA – middle panels) and 7 hepatocellular carcinoma (HCC – lower panels) patients. Violin plots illustrate the absolute abundance of sequences detected using snoRT + snoRNA primers (left panels) and the relative distribution of 3′-snoRTs, FL-snoRTs and snoRNAs (right panels) of each analyzed snoRNA sequences.

Circulating snoRNAs have been investigated as cancer biomarkers in liquid biopsies. Most of the available studies in this area focused on lung cancer. Specifically, SNORD33, SNORD66 and SNORD76 were found to be upregulated in the plasma of NCLSC patients [33,34]. SNORD89 expression was reduced in breast tissue, but it acts as an oncogene in ovarian cancer [35,36]. High expression of SNORA67 has been observed in tumors with high expression of dyskerin [37], while SNORA70E has also been implicated in the development of ovarian cancer [36]. Due to the successful detection of snoRNAs and their respective isoforms in cancer tissue, we investigated the presence of snoRNAs, 3′- and FL-snoRTs in plasma from both normal and cancer patients (unrelated to the investigated cancer specimens) via ddPCR (Fig. 5).

As for the normal plasma, the results indicated the presence of all snoRNAs and their respective isoforms in all the samples (with varying abundance) with the exception of RNF149-SNORD89 FL-snoRT. On the other hand, the analysis revealed that all three snoRNAs (SNORD89, SNORA70, and SNORA67) can be detected in the plasma of patients with HCC and BRCA. Regarding the snoRTs, RNF149-SNORD89 FL- and 3′- snoRTs were not detected in breast cancer patients' plasma, while both the FL- and 3′-snoRT were detectable in HCC patients' plasma. Although RPL10-SNORA70 3′- and FL-snoRTs were identified in all HCC and BRCA patients' plasma samples, they exhibited significant variability. Interestingly, while both EIF4A1-SNORA67 3′- and FL snoRTs were detected in HCC plasma, only the 3′-snoRT was present in breast cancer plasma, suggesting that the presence of these isoforms in plasma may be cancer-specific. These findings also suggest the need for further investigations to evaluate the potential of transcripts containing snoRNA sequences as biomarkers in liquid biopsies.

Moreover, considering the findings in cancer-derived specimens altogether, they suggest that in the presence of a very high cumulative expression of a given snoRNA, the levels of the corresponding snoRTs are generally low or undetectable, while they are relatively more abundant in association with a lower cumulative representation of the snoRNA (e.g., in the case of RNF 149-SNORD89).

## 4 Conclusions

In the present study we performed for the first time a comprehensive evaluation of snoRT using third generation transcriptomic data from cells and tissues of human origin. This analysis enabled us to identify a conservative threshold to distinguish 3′- and FL-snoRTs at least for the relevant fraction of transcripts retaining the snoRNA sequence into a coding sequence. We also demonstrated that snoRTs are widely represented in a variety of normal tissues and cancer-derived cell lines both in their FL-form and also in their shorter 3′-variant. Interestingly this latter variant for some transcripts represents the only form detected. In addition, snoRTs can frequently be localized into the cellular cytoplasmic fraction.

It is important to mention that short reads and gene based transcriptomic analysis completely overlooked these transcripts, potentially missing critical insights into their function and their involvement in cancer and possibly other diseases. Indeed, according to different studies, snoRT can play a functional role in cell and tissue homeostasis [14,16]. Finally, considering that selected snoRNAs have been previously proposed as biomarkers in different disorders [[38], [39], [40]] our results point out that a variable but sometimes consistent proportion of snoRNA sequences detected both in tissues and in liquid biopsy samples are, in fact, present as snoRTs. Altogether, these results strongly indicate that snoRTs should be further investigated in different contexts in order to better understand their biogenesis, distinct sequence, presence, and role within cells. For instance, snoRT biogenesis may reflect the different activity of the spliceosome in different cellular contexts or, on the other hand, a more specific regulation in snoRNA processing. snoRT presence and distinct sequence features definition can benefit by the application of direct RNA long-read transcriptomic analysis at high depth in tumor samples. Finally, the characterization of the role of snoRTs within the cells clearly extends beyond the scope of the current study but future investigations could explore their potential activity in guiding RNA modification in different cellular compartments, and/or in sponging regulatory elements such as RNA binding proteins or microRNAs.

## CRediT authorship contribution statement

**Guglielmo Rambaldelli:** Writing – original draft, Methodology, Investigation, Formal analysis, Conceptualization. **Sidra Asghar:** Writing – original draft, Methodology, Investigation, Formal analysis, Data curation, Conceptualization. **Giulia Venturi:** Writing – original draft, Methodology, Investigation, Formal analysis, Data curation, Conceptualization. **Federico Zacchini:** Writing – original draft, Methodology, Investigation, Formal analysis, Data curation, Conceptualization. **Margherita Serra:** Writing – review & editing, Resources, Methodology. **Catia Giovannini:** Writing – review & editing, Resources, Methodology. **Laura Gramantieri:** Writing – review & editing, Resources, Methodology. **Marco Bernini:** Writing – review & editing, Resources, Methodology. **Alberto Inga:** Writing – review & editing, Conceptualization. **Erik Dassi:** Writing – review & editing, Conceptualization. **Lorenzo Montanaro:** Writing – review & editing, Supervision, Funding acquisition, Conceptualization.

## Ethical approval

The study was approved by the local ethical committee, The Central Emilia Wide Area Ethical Committee of the Emilia-Romagna Region (CE-AVEC) (EM347-2023_140/2019/Sper/AOUBo and 528/2021/Sper/AOUBo). Informed consent was obtained from each participant.

## Code availability

The in-house pipeline for snoRT's identification and categorization is available on GitHub at: https://github.com/Rambaldelli/snoRTer.

## Funding

This work was funded by the 10.13039/501100003196Italian Ministry of Health [RC-2024-2790136 to L.M.]; The Italian Ministry for University and Research – PRIN2022 [2022R8R3LB to L.M.] and Fondazione AIRC per la ricerca sul cancro (grant number IG 30616 to L.M.]. G.V. was supported by an AIRC fellowship for Italy [grant number fellowships Italy post-doc 28136].

## Declaration of competing interest

The authors declare that they have no known competing financial interests or personal relationships that could have appeared to influence the work reported in this paper.

## Abbreviations

snoRNAs: Small nucleolar RNAs
snoRTs: snoRNA retaining transcripts
SNORD: C/D box snoRNAs
FBL: Methyltransferase fibrillarin
SNORA: H/ACA box snoRNAs
Ψ: Pseudouridine
DKC1: Dyskerin
rRNA: Ribosomal RNA
lncRNAs: Long non coding RNA
SNHG: Small nucleolar RNA host genes
FL-snoRT: Full-length snoRNA retaining mRNA isoforms
3′-snoRT: 3′ polyadenylated fragments terminating at their 5′ in proximity to the snoRNA retaining sequence
ONT: Oxford Nanopore Technology
BRCA: Breast cancer
HCC: Hepatocellular carcinoma
ddPCR: Droplet Digital PCR

## Appendix A Supplementary data

Fig. S1 Intron Retention in Tissue: Intron retention frequency comparison in healthy tissue. The dataset containing BRCA and HCC samples parameters is available at the following link 10.5281/zenodo.14989084 Fig. S1

Supplementary Table 1 Patients: Clinical data Supplementary Table 1

Supplementary Table 2 Primers: Sequences used for ddPRC. Supplementary Table 2

Supplementary Table 3 Manually annotated snoRTs Supplementary Table 3

Supplementary Table 4 SnoFL-3: Raw counts for FL- and 3′-snoRTs Supplementary Table 4
