# Corrigendum to < ‘Characterization of small nucleolar RNA retaining transcripts in human normal and cancer cells’> <[Non-coding RNA Research vol. 13 (2025) 153–161]>

**DOI:** 10.1016/j.ncrna.2026.01.002

**Published:** 2026-01-19

**Authors:** G. Rambaldelli, S. Asghar, G. Venturi, F. Zacchini, M. Serra, C. Giovannini, L. Gramantieri, M. Bernini, A. Inga, E. Dassi, L. Montanaro

**Affiliations:** a Department of Medical and Surgical Sciences (DIMEC), Bologna University, Via Massarenti 9, 40138, Bologna, Italy; b Departmental Program in Laboratory Medicine, IRCCS Azienda Ospedaliero-Universitaria di Bologna, Via Albertoni 15, 40138, Bologna, Italy; c Unit of Breast Surgery, IRCCS Azienda Ospedaliero-Universitaria di Bologna, Via Albertoni 15, 40138, Bologna, Italy; d Division of Internal Medicine, Hepatobiliary and Immunoallergic Diseases, IRCCS Azienda Ospedaliero-Universitaria di Bologna, Via Albertoni 15, 40138, Bologna, Italy; e Department of Cellular, Computational and Integrative Biology (CIBIO), Trento University, Via Sommarive 9, Povo, 38123, (TN), Italy

The authors regret the following text was not included in the Acknoledgments section < The authors are grateful to the Genotype-Tissue Expression (GTEx) Project for access to the human normal tissue ONT sequencing dataset. GTEx Project was supported by the Common Fund of the Office of the Director of the National Institutes of Health (commonfund.nih.gov/GTEx). Additional funds were provided by the NCI, NHGRI, NHLBI, NIDA, NIMH, and NINDS. Donors were enrolled at Biospecimen Source Sites funded by NCI\Leidos Biomedical Research, Inc. subcontracts to the National Disease Research Interchange (10XS170), Roswell Park Cancer Institute (10XS171), and Science Care, Inc. (X10S172). The Laboratory, Data Analysis, and Coordinating Center (LDACC) was funded through a contract (HHSN268201000029C) to the The Broad Institute, Inc. Biorepository operations were funded through a Leidos Biomedical Research, Inc. subcontract to Van Andel Research Institute (10ST1035). Additional data repository and project management were provided by Leidos Biomedical Research, Inc. (HHSN261200800001E). The Brain Bank was supported supplements to University of Miami grant DA006227. Statistical Methods development grants were made to the University of Geneva (MH090941 & MH101814), the University of Chicago (MH090951,MH090937, MH101825, & MH101820), the University of North Carolina - Chapel Hill (MH090936), North Carolina State University (MH101819),Harvard University (MH090948), Stanford University (MH101782), Washington University (MH101810), and to the University of Pennsylvania (MH101822). The datasets used for the analyses described were obtained from dbGaP at http://www.ncbi.nl>.

The authors would like to apologise for any inconvenience caused.

