# Supplementary figures and images for: Characterization of small nucleolar RNA retaining transcripts in human normal and cancer cells
Source: Noncoding RNA Res. 2025 May 9;13:153–61. doi: 10.1016/j.ncrna.2025.05.004 (PMC12152556; doi:10.1016/j.ncrna.2025.05.004)

# Intron Retention Frequency

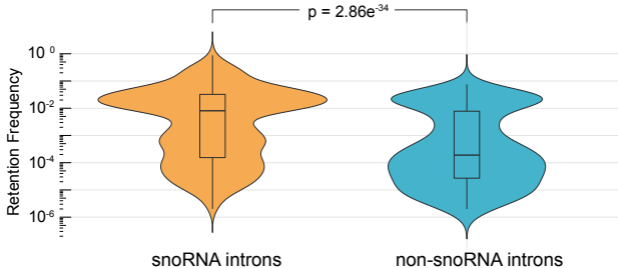

Supplement: Fig. S1 — Intron Retention in Tissue: Intron retention frequency comparison in healthy tissue. The dataset containing BRCA and HCC samples parameters is available at the following link 10.5281/zenodo.14989084 [file mmc1.pdf]
